# Wound Dehiscence after Posterior Sagittal Anorectoplasty in Children with Anorectal Malformations

**DOI:** 10.1155/2018/2930783

**Published:** 2018-11-11

**Authors:** Louise Tofft, Martin Salö, Einar Arnbjörnsson, Pernilla Stenström

**Affiliations:** Department of Paediatric Surgery, Skåne University Hospital, Department of Clinical Sciences, Paediatrics, Lund University, Lund, Sweden

## Abstract

**Aim of the Study:**

To assess the frequency of and identify contributing factors to wound dehiscence after posterior sagittal anorectoplasty (PSARP) in children born with anorectal malformations (ARM).

**Methods:**

Ethical approval was obtained (DNR 2017/191). Charts of all children with anorectal malformations (ARM) reconstructed with PSARP, limited PSARP, or PSARVUP at a tertiary centre of paediatric surgery between 2001 and 2016 were reviewed. Wound dehiscence within 30 days postoperatively was analysed regarding gender, prematurity, birth weight, type of ARM, other congenital malformations, single- or multistaged reconstruction, age and weight at reconstruction, postoperative antibiotics, and fasting. Multiple regression analysis was performed for risk factors in single-stage PSARP or limited PSARP, presented as odds ratio (OR) with 95% confidence interval (CI).

**Main Results:**

Ninety patients were included, of which 53 (59%) were males. Single-staged PSARP was performed in 40 (44%) patients and 50 (56%) had a multistaged reconstruction with a colostomy. Wound dehiscence was significantly more common among patients without a colostomy; 17 (43%) vs. 11 (22%) (p=0.043). In patients with single-stage PSARP, no single factor was identified to increase the risk for wound dehiscence: cardiac malformations (OR 3.73) (95% CI 0.78-17.88), low weight at surgery (OR 1.56) (95% CI 0.36-6.99), antibiotics < 1 day (OR 1.6) (95% CI 0.43-5.94), or short fasting 0-3 days (OR 4.44) (95% CI 0.47-42.18).

**Conclusions:**

A divided colostomy protected against wound dehiscence after PSARP. No risk factor for wound dehiscence after single-staged PSARP was identified. Further studies are needed to establish contributing factors to uncomplicated wound healing after PSARP.

## 1. Introduction

Anorectal malformations (ARM) are a group of congenital malformations involving the anorectum and pelvic floor affecting 1/5000 of live births [1]. The anomalies are classified according to the Krickenbeck International classification [2] and they vary in severity and functional prognosis [3].

Since the 1980s, ARMs are reconstructed predominately through the posterior sagittal anorectoplasty (PSARP). The reconstructive surgery is conducted through a single-stage procedure or a multiple-stage procedure with a colostomy depending on the type of ARM [4, 5]. A protective colostomy is recommended in severe types of ARM in order to facilitate surgery and decrease the risk of postoperative complications. It is still under debate if the stoma should be divided or diverted, and if a diversion of stool prevents complications in vestibular fistulas [5–8]. Postoperative complications are known to increase morbidity and patient suffering and to consume health care resources [9, 10]. Prevention of postoperative complications after PSARP is of importance in order to avoid a prolonged time in hospital, pain, and need for secondary operations [5, 8].

Previous reports on postoperative complications after PSARP have focused mainly on damage to the urinary tract and recurrent fistulas [11–13]. Use of antibiotic prophylaxis and postoperative fasting are factors reported to influence the risk of overall complications after PSARP [8]. Studies on short-term complications after PSARP, such as wound dehiscence, are sparse as are possible long-term effects of such wound complications [6, 7, 14, 15]. The prevalence of wound complications in ARM and risk factors for them has not been fully investigated despite the probable relevance for patient morbidity, final outcome, and health care economy.

Therefore, the main purpose of this study was to assess the frequency of wound dehiscence after PSARP at a tertiary unit of paediatric surgery. The secondary aims were to identify possible risk factors and protective factors for wound dehiscence after single-stage PSARP.

## 2. Materials and Methods

### 2.1. Study Design

The study was a retrospective chart study with analysis of postoperative complications. The medical charts of all patients with ARM treated at a tertiary centre of paediatric surgery from 2001 through 2016 were reviewed. All patients who underwent either a single-stage or a multistage surgical correction through PSARP, limited PSARP, or PSARVUP were included. Patients who died before PSARP, had their surgical correction elsewhere, had not yet undergone surgical correction at the time of the study, or had anal stenosis that only required dilatation were excluded.

The included patients' charts were evaluated concerning gestational age, birth weight, type of anorectal malformation according to the Krickenbeck classification, associated malformations, and associated syndromes. Associated malformations included congenital anomalies of the gastrointestinal tract, urinary tract, genitalia, heart, spine, spinal cord, and limbs. Operative data concerning single- or multistage reconstructive surgery, type of reconstructive surgery, and patients' age and weight at time of surgery were noted. Further, data concerning perioperative routines, including time of postoperative fasting and use of antibiotics, were collected. PSARP-related complications data such as wound infection, wound dehiscence, sepsis, and urinary tract infection up to 30 days postoperatively were collected. Wound dehiscence was defined as superficial (only skin rupture) or deep (subdermal structures involved). All surgical complications were graded according to the Clavien-Dindo classification [9].

### 2.2. Surgical Management

Preoperative assessment determining type of ARM included colostogram, contrast examination of the cystourinary tract, cystoscopy, clinical examination under general anaesthesia, and, whenever needed, MRI of the pelvic floor. According to the local programme for ARM, all patients underwent routine investigations concerning cardiac, urinary tract, vertebral, and spinal malformations by plain X-ray and ultrasonography. Prior to 2007, assessment of spinal malformations was performed following clinical signs of pathology or symptoms. From 2007, the screening was performed with ultrasonography and a subsequent MRI in case of any suspected spinal malformation. Associated syndromes were diagnosed through chromosomal and genetic testing.

Divided colostomies were used in multistaged approaches. All ARMs were reconstructed through PSARP or limited PSARP in cases of perineal fistulas, with no supralevator dissection, except for cloacal malformations which were reconstructed through the posterior sagittal anorectovagino-urethroplasty (PSARVUP). During PSARP, a guide wire was placed endoscopically through the fistula from the rectum to the urinary tract to facilitate identification of the fistula during dissection of the pelvic floor [16]. Antibiotic prophylaxis was given in one dose intravenously: either cefuroxime 20 mg/kg or Eusaprim® (sulfamethoxazole + trimethoprim) according to age, plus metronidazole 20 mg/kg preoperatively as routine, and postoperatively according to clinical evaluation as a result of the decision by the responsible surgeon. Wound management was heterogeneous with sodium chloride or water and soap cleansing and use of multiple kinds of dressings. No topical antibiotics were used.

Microbiological cultivation of postoperative wounds and postoperative treatment with antibiotics, administered orally or intravenously, or reoperation with creation of a protective stoma was dependent upon the decision by the responsible surgeon after clinical evaluation.

### 2.3. Statistical Analysis

Descriptive data analyses and group comparisons were performed using Excel (Microsoft® Excel for Mac, version 15.39, 2017) and SPSS® (IBM® SPSS® Statistics, version 24, 2016). In group comparisons for dichotomous data, Fisher's exact test was used while Mann-Whitney U-test was used for continuous parameters. To evaluate risk factors for wound dehiscence, multivariate logistic regression was used and presented as odds ratios (OR) with a 95% confidence interval (CI). A p-value < 0.05 was considered to be statistically significant.

### 2.4. Ethics

Clinical follow-ups of patients with ARM, including patients of this particular study, were approved by the local ethics committee (DNR 2017/191).

## 3. Results

### 3.1. Patients

**Figure 1:**
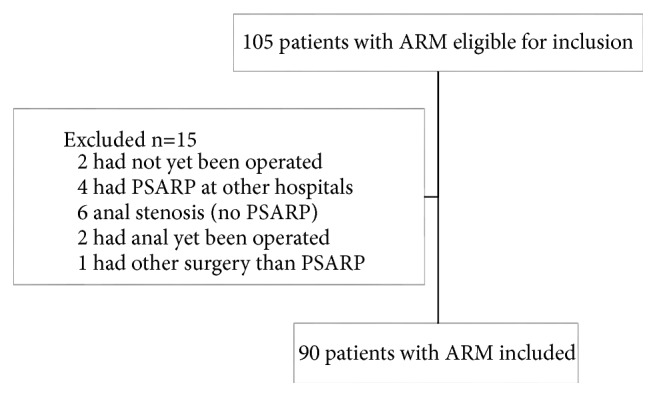
Fallout chart of reviewed medical charts of patients with ARM at a tertiary centre of paediatric surgery 2001-2016.

A total of 90 patients reconstructed with PSARP, limited PSARP, or PSARVUP were included (Figure 1).  Table 1 displays details of the background data of the children. The majority of patients were boys and the most common ARM-subtype in both genders was the perineal fistula. The majority of patients were born at full time but a quarter were small for gestational age and the vast majority had at least one other concomitant malformation. Fifty patients (56%) had either a PSARP or PSARVUP through a multistaged reconstruction with a colostomy. Forty patients (44%) had a single-staged reconstruction where patients with perineal fistulas constituted the great majority. The patients with a single-stage reconstruction were younger and lighter in weight than patients with stomas at the time of PSARP. There was no difference concerning prolonged antibiotic prophylaxis after single- or multistaged PSARP (Table 1).

### 3.2. Complications

Overall, wound dehiscence occurred in 28 (31%) patients of whom 19 (21%) had a superficial dehiscence (only skin) and nine (10%) a deep dehiscence. Wound dehiscence occurred to a significantly lesser degree among patients with a colostomy (Table 2).

The severity of wound complications according to the Clavien-Dindo classification system for postoperative complications within 4 weeks is displayed in Table 2. No patient with wound dehiscence had any reoperation of the neoanus, but one patient, who did not have any postoperative infection or dehiscence, underwent a reoperation 10 years later due to a ventrally misplaced neoanus. Other postoperative complications within 4 weeks postoperatively, besides the perineal conditions, were urinary tract infection in five (6%) patients (all in the multistage group) and sepsis in three (3%) patients (two cases in the multistage group and one case in the single-stage group).

Bacterial cultivation was performed in nine of the dehiscence cases and showed growth of* Enterococcus* species,* E-coli, *and* Staphylococcus* species. All wound dehiscence cases occurred within 14 days postoperatively. Another three patients (3%) were treated due to a suspected perineal surgical site infection without any wound dehiscence.

The wound dehiscence group showed no statistical difference regarding gender, smallness for gestational age, concomitant heart malformation, weight at time of reconstruction, or VACTERL association compared to the nondehiscence group (Table 3).

### 3.3. Risk Factors for Wound Dehiscence in Single-Stage PSARP

In the multivariate risk analysis regarding wound dehiscence after single-stage PSARP or limited PSARP, no risk factor for wound dehiscence could be identified (Table 4).

## 4. Discussion

Wound dehiscence after PSARP at this tertiary centre was present in 31% of all cases and among patients reconstructed through single-stage PSARP dehiscence that occurred in 43%. Wound dehiscence occurred statistically less frequently in multistage PSARP in presence of a colostomy compared to single-stage reconstruction. The study revealed no proven risk factor among the studied demographical or surgically related factors regarding single-staged PSARP.

The wound dehiscence rate overall was found to correspond with some previous reports where 0–40% dehiscence rates are reported after single-staged PSARP [6, 7, 11, 12, 14, 15]. In those reports, risk factors were not analysed, nor were the circumstances for postoperative wound treatment reported in comparable ways.

A colostomy has previously been acknowledged to protect the surgical site after PSARP from postoperative infections [1], a finding that was borne out by this study. Theoretically, this could be because the colostomy prevents faecal bacteria from entering the perineal wound. However, this study also indicated that stoma formation did not prevent wound dehiscence in all cases and that there may be other influencing factors. The consequences for patients undergoing a multistaged approach need to be considered. Although a colostomy protects against wound dehiscence, these patients need multiple surgeries with associated potential complications [17].

Which surgical site treatment to be preferred in order to prevent wound complications after PSARP needs to be further explored and documented. Previous studies of surgical site infections of other locations have listed the use of monofilament sutures and topical antibiotics as effective preventive measurements [10]. During this study, we made informal enquiries to other well-established centres in the world treating ARM and asked for their local wound management programme. Several answered that they used topical antibiotics after PSARP. Such treatment is not recommended in our country, due to risk of antibiotic resistance. To the best of the authors' knowledge, there is no published study on effects and outcome after use of topical antibiotics in wound management after PSARP. In that setting, we would also like to highlight another interesting and possible potential contributing factor of successful wound healing: the microbiota of the gut which is a fairly new and largely unknown area of research [18, 19]. Its implications for postoperative wound healing after PSARP could be worth exploring in the future.

The strength of this study is that the collection of data could be assured by the use of a stable and complete local ARM register as well as detailed medical charts over several years. At our centre, children with ARM are followed up rigorously and, therefore, reports of complications are trustworthy. All complications have been documented on a regular basis and therefore the honesty of reported complications must be acknowledged. The first author has not been involved in the surgical management of the patients and thus has been able to review the charts in an impartial way. The limitations might be attributed to the retrospective design, including a fairly high rate of missing data regarding weight at surgery due to a change of digital medical chart systems and to the limited size of the study population with secondary low power to reach statistical significances.

Although this study revealed a fairly high incidence of wound complications, and even though the reasons for it are not completely understood, it highlights the importance of transparency regarding postoperative complications in order to improve outcome after PSARP. The results could be used to design future studies aimed at unveiling contributing factors of wound dehiscence after PSARP.

## 5. Conclusions

Wound dehiscence was less frequent in multistage PSARP using a divided colostomy. No risk factor for wound dehiscence after single-stage PSARP was identified. Further studies are needed to establish contributing factors for uncomplicated wound healing after PSARP.

## Figures and Tables

**Table 1:** Patients with ARM reconstructed with PSARP, limited PSARP, or PSARVUP.

	**All patients**	**Multistaged reconstruction**	**Single-staged reconstruction**	**P value** ^∗^
**Total**	90 (100)	50 (56)	40 (44)	
**Gender**				
Male	53 (59)	31 (62)	22 (55)	0.53
Female	37 (41)	19 (38)	18 (45)	
**ARM subtype**				
Perineal (M/F)	24 (45) / 17 (46)	3 (6)	38 (94)	
Vestibular	15 (41% of F)	14 (28)	1 (3)	
Rectourethral	20 (38% of M)	19 (38)	1 (3)	
Rectovesical	4 (8% of M)	4 (8)	0	
Cloaca	4 (11% of F)	4 (8)	0	
No fistula (M/F)	5 (9) / 1 (2)	6 (12)	0	
**Prematurity **n=77^∗∗∗^	15 (19)	11 (24)	4 (13)	0.25
**SGA **n=80^∗∗∗^	23 (29)	16 (33)	7 (22)	0.32
**Concomitant malformations**				
Total (at least one)	63 (70)	44 (88)	19 (48)	*<0.001*
Cardiac n=84^∗∗∗^	28 (33)	18 (38)	10 (36)	0.48
Urinary tract n=85^∗∗∗^	25 (29)	20 (42)	5 (14)	*0.008*
Other skeletal	19 (21)	14 (28)	5 (13)	0.12
Vertebral n=60^∗∗∗^	30 (50)	25 (64)	5 (24)	*0.003*
Spinal cord n=47^∗∗∗^	14 (30)	10 (29)	4 (31)	0.93
Genital	17 (19)	14 (28)	3 (8)	*0.015*
Gastrointestinal tract	12 (13)	9 (18)	3 (8)	0.21
VACTERL association	24 (27)	19 (38)	5 (13)	*0.008*
**Concomitant syndromes**				
Total	15 (17)	10 (20)	5 (13)	0.40
Trisomy 21	6 (7)	5 (10)	1 (3)	0.22
**Age at PSARP **(days)		122 (8–1234)	2 (0-616)	*<0.001* ^∗∗^
**Weight at PSARP **(g) n=62^∗∗∗^		5700 (3100–14000)	3500 (1600–11400)	*<0.001* ^∗∗^
**Antibiotic prophylaxis **> 1 day	33 (37)	19 (38)	14 (35)	0.83

Values presented as the absolute number and percentage of patients, n (%), and as median (min–max); ARM: anorectal malformations, PSARP: posterior sagittal anorectoplasty, PSARVUP: posterior sagittal anorectal vaginal urethroplasty; M: male, F: female; Prematurity: GW< 37; SGA: small for gestational age; ^∗^Fisher's Exact test two-tailed, ^∗∗^Mann Whitney U-test two-tailed; ^∗∗∗^number of patients with available data

**Table 2:** Wound dehiscence classified according to Clavien-Dindo in 90 patients reconstructed with PSARP, limited PSARP, or PSARVUP.

	**Multistaged reconstruction**	**Single-staged reconstruction**	**P value** *∗*
**Total**	50	40	
**Wound dehiscence**	11 (22)	17 (43)	*0.043*
**Clavien-Dindo**			
(1) No treatment	2 (4)	5 (12.5)	
(2) Medically treated	9 (18)	8 (20)	
(3a) Examined under general anaesthesia	0 (0)	1 (2.5)	
(3b) Stoma establishment	0 (0)	3 (8)	
(4) Intensive care	0 (0)	0 (0)	
(5) Death	0 (0)	0 (0)	

Values presented as the absolute number and percentage of patients, n (%); PSARP: posterior sagittal anorectoplasty, PSARVUP: posterior sagittal anorectal vaginal urethroplasty; *∗* Fisher Exact test two-tailed.

**Table 3:** Demographical factors for wound dehiscence in 90 patients reconstructed with PSARP, limited PSARP, or PSARVUP.

	**Wound dehiscence**	**No wound dehiscence**	**P value** ^∗^
**Total**	28	62	
**Gender female**	11 (39)	26 (42)	1
**SGA **n=80^∗∗∗^	7 (28)	16 (29)	0.92
**Cardiac malformation **n=84^∗∗∗^	9 (33)	19 (33)	1
**VACTERL association**	6 (21)	18 (29)	0.608
**Weight at surgery **(g) n=62^∗∗∗^	3800	5000	0.327^∗∗^
	(2200-9400)	(1600-14000)	
**Colostomy**	11 (39)	39 (63)	*0.043*

Values presented as the absolute number and percentage of patients, n (%), and as median (min–max); PSARP: posterior sagittal anorectoplasty, PSARVUP: posterior sagittal anorectal vaginal urethroplasty; SGA: small for gestational age; ^∗^Fisher's exact test two-tailed, ^∗∗^Mann-Whitney U-test two-tailed; ^∗∗∗^number of patients with available data.

**Table 4:** Multivariate logistic regression analysis of risk factors for wound dehiscence in 40 patients reconstructed with single-staged PSARP or limited PSARP.

	**Single-staged reconstruction**	**Wound dehiscence**	**OR (95**%** CI)**	**P value**
**Total**	40	17 (43)		
**Gender female**	18 (45)	6 (35)	0.50 (0.14–1.81)	0.348
**Cardiac malformation **	10 (25)	7 (41)	3.73 (0.78–17.88)	0.066
**Weight at surgery **<3500g	15 (38)	5 (29)	1.56 (0.36–6.69)	0.716
**Antibiotics **<1 day	26 (65)	10 (59)	1.60 (0.43–5.94	0.521
**Fasting **0–3 days	34 (85)	16 (94)	4.44 (0.47–42.18)	0.216

Values presented as the absolute number and percentage of patients, n (%); PSARP: posterior sagittal anorectoplasty; OR: odds ratio, CI: confidence interval.

## Data Availability

The data is available but not for open publication.

## References

[1] Levitt M. A., Peña A. (2007). Anorectal malformations. *Orphanet Journal of Rare Diseases*.

[2] Holschneider A., Hutson J., Peña A. (2005). Preliminary report on the International Conference for the Development of Standards for the Treatment of Anorectal Malformations. *Journal of Pediatric Surgery*.

[3] Stenström P., Kockum C. C., Emblem R., Arnbjörnsson E., Bjørnland K. (2014). Bowel symptoms in children with anorectal malformation - A follow-up with a gender and age perspective. *Journal of Pediatric Surgery*.

[4] Peña A., Devries P. A. (1982). Posterior sagittal anorectoplasty: Important technical considerations and new applications. *Journal of Pediatric Surgery*.

[5] Bischoff A., Levitt M. A., Peña A. (2013). Update on the management of anorectal malformations. *Pediatric Surgery International*.

[6] Elsaied A., Aly K., Thabet W., Magdy A. (2013). Two-stage repair of low anorectal malformations in girls: Is it truly a setback?. *Annals of Pediatric Surgery*.

[7] Karakus S. C., User I. R., Akcaer V., Ceylan H., Ozokutan B. H. (2017). Posterior sagittal anorectoplasty in vestibular fistula: with or without colostomy. *Pediatric Surgery International*.

[8] Morandi A., Ure B., Leva E., Lacher M. (2015). Survey on the management of anorectal malformations (ARM) in European pediatric surgical centers of excellence. *Pediatric Surgery International*.

[9] Dindo D., Demartines N., Clavien P. (2004). Classification of surgical complications: a new proposal with evaluation in a cohort of 6336 patients and results of a survey. *Annals of Surgery*.

[10] Alexander J. W., Solomkin J. S., Edwards M. J. (2011). Updated recommendations for control of surgical site infections. *Annals of Surgery*.

[11] Chan K. W. E., Lee K. H., Wong H. Y. V. (2014). Outcome of patients after single-stage repair of perineal fistula without colostomy according to the Krickenbeck classification. *Journal of Pediatric Surgery*.

[12] Kuijper C. F., Aronson D. C. (2010). Anterior or posterior sagittal anorectoplasty without colostomy for low-type anorectal malformation: How to get a better outcome?. *Journal of Pediatric Surgery*.

[13] Bischoff A., Peña A., Levitt M. A. (2013). Laparoscopic-assisted PSARP - The advantages of combining both techniques for the treatment of anorectal malformations with recto-bladderneck or high prostatic fistulas. *Journal of Pediatric Surgery*.

[14] Kumar B., Kandpal D. K., Sharma S. B., Agrawal L. D., Jhamariya V. N. (2008). Single-stage repair of vestibular and perineal fistulae without colostomy. *Journal of Pediatric Surgery*.

[15] Short S. S., Bucher B. T., Barnhart D. C. (2018). Single-stage repair of rectoperineal and rectovestibular fistulae can be safely delayed beyond the neonatal period. *Journal of Pediatric Surgery*.

[16] Stenström P., Anderberg M., Kockum C., Arnbjornsson E. (2013). Endoscopically Placed Rectourethral Guidewire Facilitates the Reconstruction of Anus in Children with Anorectal Malformations: A Case Report. *European Journal of Pediatric Surgery Reports*.

[17] Pena A., Migotto-Krieger M., Levitt M. A. (2006). Colostomy in anorectal malformations: A procedure with serious but preventable complications. *Journal of Pediatric Surgery*.

[18] Lee J. A., Chico T. J. A., Renshaw S. A. (2017). The triune of intestinal microbiome, genetics and inflammatory status and its impact on the healing of lower gastrointestinal anastomoses. *FEBS Journal*.

[19] Lederer A.-K., Pisarski P., Kousoulas L., Fichtner-Feigl S., Hess C., Huber R. (2017). Postoperative changes of the microbiome: Are surgical complications related to the gut flora? A systematic review. *BMC Surgery*.

